# Can resources moderate the impact of levels of frailty on adverse outcomes among (pre-) frail older people? A longitudinal study

**DOI:** 10.1186/s12877-017-0583-4

**Published:** 2017-08-17

**Authors:** Linda P.M. Op het Veld, Bart H.L. Ament, Erik van Rossum, Gertrudis I.J.M. Kempen, Henrica C.W. de Vet, KlaasJan Hajema, Anna J.H.M. Beurskens

**Affiliations:** 1 Centre of Research Autonomy and Participation for Persons with a Chronic Illness, Faculty of Health, Zuyd University of Applied Sciences, P.O. Box 550, 6400 AN Heerlen, The Netherlands; 2 CAPHRI, Care and Public Health Research Institute, Department of Health Services Research, Maastricht University, P.O. Box 616, 6200 MD Maastricht, The Netherlands; 3 Department of Epidemiology and Biostatistics, Amsterdam Public Health research institute, VU University Medical Center, De Boelelaan 1089A, 1081 HV Amsterdam, The Netherlands; 4 Community Health Service South Limburg, Academic Collaborative Centres Public Health (ACC), Geleenbeeklaan 2, 6166 GR Sittard-Geleen, The Netherlands; 5 CAPHRI, Care and Public Health Research Institute, Department of Family Practice, Maastricht University, P.O. Box 616, 6200 MD Maastricht, The Netherlands

**Keywords:** Frailty, Older people, Resources, Moderating factors, Mortality, Hospitalisation, Disability

## Abstract

**Background:**

Higher levels of frailty result in higher risks of adverse frailty outcomes such as hospitalisation and mortality. There are, however, indications that more factors than solely frailty play a role in the development of these outcomes. The presence of resources, e.g. sufficient income and good self-management abilities, might slow down the pathway from level of frailty to adverse outcomes (e.g. mortality). In the present paper we studied whether resources (i.e. educational level, income, availability of informal care, living situation, sense of mastery and self-management abilities) moderate the impact of the level of frailty on the adverse outcomes mortality, hospitalisation and the development of disability over a two-year period.

**Methods:**

Longitudinal data on a sample of 2420 community-dwelling pre-frail and frail older people were collected. Participants filled out a questionnaire every six months, including measures of frailty, resources and outcomes. To study the moderating effects of the selected resources their interaction effects with levels of frailty on outcomes were studied by means of multiple logistics and linear regression models.

**Results:**

Frail older participants had increased odds of mortality and hospitalisation, and had more deteriorating disability scores compared to their pre-frail counterparts. No moderating effects of the studied resources were found for the outcomes mortality and hospitalisation. Only for the outcome disability statistically significant moderating effects were present for the resources income and living situation, yet these effects were in the opposite direction to what we expected. Overall, the studied resources showed hardly any statistically significant moderating effects and the directions of the trends were inconsistent.

**Conclusions:**

Frail participants were more at risk of mortality, hospitalisation, and an increase in disability. However, we were unable to demonstrate a clear moderating effect of the studied resources on the adverse outcomes associated with frailty (among pre-frail and frail participants). More research is needed to increase insight into the role of moderating factors. Other resources or outcome measures should be considered.

## Background

In societies with growing numbers of older people, the numbers of frail older people are increasing as well, which imposes a burden on the healthcare system [1]. Despite the frequent use of the frailty concept, there is a lack of consensus regarding its nature and definition. Roughly, three approaches to conceptualise frailty emerge from the literature. One approach considers frailty to be a decline in physiological aspects of functioning. This phenotype of frailty, as described by Fried and colleagues, comprises five predefined physical frailty criteria and distinguishes between non-frail, pre-frail and frail persons [2]. A second approach considers frailty to be the accumulation of deficits across various domains (e.g. comorbidities, psychological functioning and physical functioning). Rockwood et al. proposed a Frailty Index which is often used for this approach. It is characterised by the use of a non-fixed set of clinical conditions and diseases [3, 4]. Similar to the second one, a third approach also considers frailty to be a multidimensional concept. In contrast with the accumulation of deficits approach, this third approach includes a pre-defined set of physical, social and psychological questions (e.g. Tilburg Frailty Indicator (TFI)) [5].

The variety in definitions of frailty has resulted in an abundant number of screening instruments [6, 7]. Irrespective of the instrument used, however, researchers agree that higher levels of frailty result in higher risks of adverse outcomes of frailty. For example, Fried and colleagues have shown that mortality was over threefold higher over a seven-year period for people who were frail at baseline compared to non-frail people (43% vs. 12%) [2]. The same holds for hospitalisation (96% vs 79%) and disability (63% vs 23%). There are however indications that more factors than solely frailty play a role in the development of adverse outcomes. Verbrugge and Jette proposed, in their process of disablement, a pathway that links pathology through impairments and functional limitations to disability. However, this pathway is supposed to be influenced by risk factors and particularly moderated by intra- and extra-individual factors [8] of which the latter two can be considered as resources that alleviate the impact of, for example, impairments and functional limitations on disability. Similarly, the associations between the levels of frailty and adverse outcomes may also be moderated by different resources individuals may have. In frail older people, characteristics such as low income, low educational level, and living alone status, intra-individual factors (e.g. sense of mastery, self-management abilities) and extra-individual factors (e.g. availability of informal care) may moderate the impact of frailty on the development of adverse outcomes. Although prior studies have shown that several of these factors are related to frailty [9, 10], studies on potential moderating effects of these factors (resources) between frailty level and adverse outcomes are scarce. For example, Dent and colleagues found that hospitalized frail older people with a low sense of control had an increased likelihood of 12-month mortality compared to frail people with a good sense of control [11]. Ament and colleagues showed that the impact of personal deficits, as an indicator of frailty, on receiving professional care and self-perceived health is moderated by educational level and living alone status, although the latter was only found in women [12].

Previous research often used cross-sectional data and mainly focused on just one domain of moderating factors (e.g. environmental factors or psychological factors). The present study was designed to examine whether resources (i.e. educational level, income, availability of informal care, living situation, sense of mastery and self-management abilities) moderate the impact of frailty level on the adverse outcomes mortality, hospitalisation and the development of disability over a two-year period. As Fried’s frailty criteria are most frequently used by researchers to identify frail older people, we use them in the present study as well [6]. We focused on community-dwelling pre-frail and frail persons as they have an increased risk of suffering from adverse outcomes. We hypothesised that more favourable resources (i.e. higher educational level, higher income, availability of informal care, living with someone, higher sense of mastery and better self-management abilities) slow down the pathway from level of frailty to adverse outcomes.

## Methods

A longitudinal study with a two-year follow-up period was conducted. The study was approved by the medical ethical committee of Zuyderland and Zuyd University of Applied Sciences (METC Z,12-N-129).

### Procedure and participants

Every four years, the Community Health Services in the Netherlands send an extensive general health questionnaire to a large sample of community-dwelling people. People are questioned about their health, social situation and lifestyle [13]. A total of 56,000 people (55+ years) living in Limburg, a province in the southern part of The Netherlands, received this questionnaire in the autumn of 2012. In total, 30,130 persons returned it of whom 13,521 gave permission to potentially participate in further research. Persons younger than 65 years and not frail according to Fried’s frailty criteria (see below) [2] were excluded. Questionnaires completed by the wrong person or with a significant number of missing values were also excluded. Eventually, a total of 3162 persons were eligible for the present study and received a shorter questionnaire to obtain relevant additional baseline data. Those who responded to this additional questionnaire and gave written informed consent, were included in the present study (*n* = 2420). After 6, 12, 18 and 24 months the participants received additional questionnaires comprising questions about their level of frailty, the availability of several resources, and outcome measures. People who died, moved to a nursing home or explicitly stated that they did not want to participate anymore were considered as drop-out during the study.

### Fried frailty criteria

The five frailty criteria as described by Fried and colleagues (weight loss, exhaustion, low physical activity, slowness and weakness) were used to classify the participants into non-frail, pre-frail or frail [2]. Weight loss was measured as proposed by Fried et al., with pounds being replaced by kilograms: ‘In the last year, have you lost more than 4.5 kilograms unintentionally? (i.e. not due to dieting or exercise)’. When someone answered ‘yes’, this criterion was met. As proposed by Fried et al., two questions from the Center for Epidemiologic Studies Depression (CES-D) scale were used to measure exhaustion: ‘How often did you feel that everything you did was an effort? ‘ and ‘How often did you feel that you could not get going?’ [14, 15]. Instead of the original response options ‘rarely or none of the time (<1 day)’, ‘some or a little of the time (1-2 days)’, ‘a moderate amount of the time (3-4 days)’, ‘most of the time’, the answer options ‘always’, ‘most of the time’, ‘sometimes’, ‘occasionally’, ‘never’ were used in our study. When participants answered ‘always’ or ‘most of the time’ to at least one of the two questions this criterion was met. The criterion of physical activity was measured with a slightly adjusted version of the Short Questionnaire to Assess Health-enhancing physical activity (SQUASH) [16]. Persons were asked how many times a week they spent time walking, cycling, gardening, doing odd jobs or exercising, and how much time they spent on each activity on each occasion. Kilocalories per week were calculated and compared to the cut-off values as proposed by Fried and colleagues. The criterion slowness was assessed by asking the question: ‘Can you reach the other side of the road when the light turns green at a zebra crossing?’. If the participant answered other than ‘yes, without any trouble’, this criterion was met. The criterion of weakness was assessed by asking the question: ‘Do you experience difficulties in daily life because of low grip strength?’. This is the same question as used in the TFI [5]. If the participant answered ‘yes’, this criterion was met. Based on the sum score of these five criteria (range 0–5) people were divided into three categories: non-frail (score 0), pre-frail (score 1–2) and frail (score 3–5). To investigate possible moderating effects of resources, persons who are pre-frail or frail are a relevant population, as they are at increased risk of adverse outcomes. Therefore, only pre-frail and frail people were asked to participate in the follow-up measurements of the present study.

### Resources

Resources tested for having a moderating effect on the adverse outcomes of frailty included: educational level, income, availability of informal care, living situation, sense of mastery and self-management abilities. Educational level was divided into two categories. The lower category comprises no education, completion of primary school or pre-vocational secondary education. All other levels of completed education are included in the higher category. Statistics Netherlands, an organisation that compiles statistics and publishes information about topics directly affecting the lives of Dutch citizens (such as economic growth, consumer prices and crime) [17], provided information about disposable income per person, adjusted for differences in family composition of the household. Persons were, by Statistics Netherlands, categorised into one of five groups ranging from a low to a high income. For the present study these categories were dichotomised into two, approximately equally sized, groups: low income and high income (cut-off 19,400 euro). Availability of informal care was determined using the question ‘Suppose you got the flu and you had to stay in bed for a couple of days. Is there someone who could take care of you?’ [18]. Results were dichotomised into ‘yes’ and ‘no’. Living situation was measured by asking participants how many people were present in their household. The results were dichotomised into ‘living alone’ and ‘not living alone’. Sense of mastery was measured by using the instrument developed by Pearlin and Schooler [19]. It comprises seven statements, such as ‘There is really no way I can solve some of the problems I have’. Five-point answering options range from ‘I totally agree’ to ‘I totally disagree’. Theoretical scores ranged from 7 to 35 with higher scores indicating a higher sense of mastery. Self-management abilities were measured with the short version of the Self-Management Ability Scale (SMAS-S) [20]. It consists of six three-item subscales that reflect core abilities to form the construct of self-management of well-being. It comprises statements and questions such as ‘Are you able to have friendly contacts with others?’. Final self-management scores theoretically ranged from one to six. Higher scores indicate more self-management abilities.

### Outcome measures

Mortality, hospitalisation, and (Instrumental) Activities of Daily Living ((I)ADL) disability were used as adverse outcome measures. Mortality data (yes or no) at two-year follow-up were obtained from Statistics Netherlands. Data on hospital admission and (I)ADL disability were obtained from the self-report questionnaires. For hospital admission, respondents were asked at each of the follow-up measurements whether they had been admitted to a hospital in the previous six months. Outpatient clinic visits or emergency department visits were not included. Two groups were created: persons who reported at least one admission and those who did not. The Groningen Activity Restriction Scale was used to determine the level of (I)ADL disability and was measured at baseline and after two years [21]. This questionnaire comprises 18 items, such as ‘Can you, fully independently, wash and dry your whole body?’. There are four possible answering options ranging from ‘Yes, I can do it fully independently without any difficulty’ to ‘No, I can do it only with someone’s help’. Theoretical scores range from 18 to 72. Higher scores indicate a higher level of (I)ADL disability.

### Statistical analysis

First, descriptive statistics were computed to provide an overview of the baseline characteristics of the total study population and pre-frail and frail persons separately. Second, analyses were performed to study the main effects between levels of frailty (pre-frail used as reference standard) and the adverse outcomes adjusted for age and gender, but without taking the potential moderating effect of resources into account. Logistic regression analyses were performed for the outcomes hospitalisation and mortality. An independent samples t-test was conducted to compare the change scores between pre-frail and frail persons for the outcome disability. The third step in the analyses was to study the potential moderating effects of the resources. This was done by adding an interaction term of frailty with the specific resource in logistic (for the outcomes hospitalisation and mortality) and linear (for the outcome disability) regression models. Subsequently, regression analyses were performed for the outcomes hospitalisation and mortality with results split by resource (e.g. low and high income) to show possible differences in Odds Ratio (OR). For the outcome measure disability (at two-year follow-up) baseline disability was included as a covariate in all models. Mean change scores including standard deviations of disability were calculated to compare pre-frail and frail persons, and results were again split by resource. Scores of mastery and self-management were dichotomised, based on median values, as suggested in previous research [22]. Age and gender were added to all regression models as covariates. *P* values <0.05 were considered statistically significant. All statistical analyses were performed with IBM SPSS Statistics for Windows version 22.

## Results

A total of 2420 persons participated in our study. Their baseline characteristics are displayed in Table 1. Mean age was 76.3 ± 6.6 years and there were more female participants (60.5%) compared to males. Frailty was present in 22.2% of the study population, 77.8% were pre-frail. Pre-frail and frail participants differed statically significant in all characteristics, except for the availability of informal care (*p* = 0.185). Frail participants had worse baseline disability scores ((I)ADL disability 43.0 ± 11.8 vs 28.6 ± 10.0) and less potentially beneficial resources compared to pre-frail participants (e.g. high educational level 21.4% vs 33.9%).

**Table 1:** Baseline descriptive characteristics of the total study population, and pre-frail and frail participants separately

	Total group	Pre-frail	Frail	*P* value
	*n* = 2420	*n* = 1883 (77.8%)	*n* = 537 (22.2%)	
Age (mean ± SD)	76.3 ± 6.6	75.8 ± 6.5	78.0 ± 6.8	P < 0.001^a^
Male gender	957 (39.5%)	767 (40.7%)	190 (35.4%)	*P* = 0.025^b^
(I)ADL disability (18–72)^d^ (mean ± SD)	31.8 ± 12.0	28.6 ± 10.0	43.0 ± 11.8	P < 0.001^a^
Resources				
Level of education				
low	1579 (68.9%)	1182 (66.1%)	397 (78.6%)	*P* < 0.001^b^
high	714 (31.1%)	606 (33.9%)	108 (21.4%)	
Income				
low	1145 (47.4%)	830 (44.1%)	315 (58.8%)	*P* < 0.001^b^
high	1272 (52.6%)	1051 (55.9%)	221 (41.2%)	
Informal care				
not available	224 (9.4%)	167 (8.9%)	57 (10.9%)	*P* = 0.185^b^
available	2167 (90.6%)	1699 (91.1%)	468 (89.1%)	
Living situation				
living alone	906 (39.2%)	668 (37.0%)	238 (46.9%)	*P* < 0.001^b^
not living alone	1404 (60.8%)	1135 (63.0%)	269 (53.1%)	
Mastery (7–35)^d^ (mean ± SD)	23.1 ± 5.9	24.2 ± 5.6	19.5 ± 5.5	P < 0.001^a^
low	1093 (49.9%)	726 (42.5%)	367 (75.8%)	*P* < 0.001^b^
high	1098 (50.1%)	981 (57.5%)	117 (24.2%)	
Self-management (1–6)^d^ (mean ± SD)	3.8 ± 0.7	3.9 ± 0.7	3.5 ± 0.8	*P* < 0.001^c^
low	1045 (47.1%)	713 (41.3%)	332 (66.9%)	*P* < 0.001^b^
high	1176 (52.9%)	1012 (58.7%)	164 (33.1%)	

All results are presented as number of cases (percentage) unless stated differently

^a^Mann-Whitney U test, ^b^ Chi-square, ^c^ Independent samples t-test, ^d^ Preferable score is underlined

During the two-year follow-up 182 participants (7.5%) died and 836 participants(34.5%) were admitted to a hospital at least once. Mean disability score at two-year follow-up was 32.9 ± 12.5, while for these persons the disability score at baseline was 29.9 ± 11.0 (*p* < 0.001).

Table 2 presents the results of logistic regression analyses for mortality and hospitalisation, presenting the relation with frailty (the OR of frail versus pre-frail participants) for each level of the resources studied, including the *p* values of the interaction terms. Results for disability are presented in Table 3, displaying mean change scores of disability for pre-frail and frail participants within the resource categories, and *p* values of the interaction terms. Both in Tables 2 and 3 the first level of each resource presented is considered to be disadvantageous, the second to be beneficial.

**Table 2:** Association between frailty and outcome variables mortality and hospitalisation, within each level of the resources

	Mortality	Hospitalisation
	OR of frailty (95% CI)^a^	OR of frailty (95% CI)^a^
Frailty (frail vs. pre-frail)	2.99 (2.17–4.13)	2.21 (1.73–2.82)
Resources		
Level of education		
low	2.80 (1.85–4.25)	1.98 (1.49–2.65)
high	3.48 (1.98–6.10)	3.04 (1.80–5.13)
interaction (*p* value)	0.616	0.148
Income		
low	3.48 (2.20–5.51)	2.55 (1.81–3.58)
high	2.62 (1.64–4.21)	1.70 (1.18–2.44)
interaction (*p* value)	0.394	0.138
Informal care		
not available	1.17 (0.29–4.74)	3.18 (1.42–7.12)
available	3.12 (2.23–4.37)	2.15 (1.66–2.78)
interaction (*p* value)	0.278	0.299
Living situation		
living alone	3.08 (1.85–5.14)	2.60 (1.75–3.88)
not living alone	2.86 (1.84–4.44)	2.06 (1.49–2.85)
interaction (*p* value)	0.958	0.290
Mastery		
low	3.04 (1.96–4.73)	2.15 (1.56–2.96)
high	3.33 (1.79–6.18)	1.81 (1.10–2.97)
interaction (*p* value)	0.689	0.723
Self-management		
low	2.24 (1.47–3.42)	2.41 (1.72–3.39)
high	3.75 (2.14–6.58)	1.84 (1.22–2.78)
interaction (*p* value)	0.151	0.329

All models are adjusted for age and gender

The first level of each resource is considered disadvantageous, the second beneficial

^a^OR (95% confidence interval)

**Table 3:** Mean change in disability scores for pre-fail and frail participants, within each level of resources

	Disability	
	mean Δ disability (SD)^a^	
	pre-frail	frail
Frailty	2.82 (6.78)	3.93 (8.26)
Resources		
Level of education		
low	3.01 (6.94)	4.00 (8.11)
high	2.43 (6.38)	4.10 (7.16)
interaction (*p* value)	0.486	
Income		
low	3.01 (7.14)	2.99 (8.43)
high	2.71 (6.39)	5.18 (7.88)
interaction (*p* value)	0.002	
Informal care		
not available	3.77 (7.31)	3.48 (11.35)
available	2.68 (6.66)	3.95 (7.86)
interaction (*p* value)	0.110	
Living situation		
living alone	3.16 (7.04)	3.40 (7.96)
not living alone	2.64 (6.59)	4.72 (8.08)
interaction (*p* value)	0.011	
Mastery		
low	3.74 (7.40)	4.28 (8.36)
high	2.36 (6.03)	1.78 (6.38)
interaction (*p* value)	0.222	
Self-management		
low	3.24 (7.03)	4.26 (8.73)
high	2.53 (6.46)	2.91 (6.59)
interaction (*p* value)	0.666	

All models are adjusted for age and gender

The first level of each resource is considered disadvantageous, the second beneficial

^a^Mean change in disability score (two year follow-up –baseline) (standard deviation)

Overall, frail participants had a threefold increased risk of mortality (OR = 2.99, 95% CI = 2.17–4.13) and an over twofold increased risk of hospitalisation (OR = 2.21, 95% CI = 1.73–2.82) compared to pre-frail participants. They also deteriorated significantly faster on disability: on average 3.93 (± 8.26) points versus 2.82 (± 6.78) points for the pre-frail participants over the two-year period (*p* = 0.022).

None of the interaction terms were statistically significant for the outcomes mortality and hospitalisation, indicating no moderating effect for any of the resources, even though OR estimates sometimes differed substantially. For example, among participants with *no* availability of informal care frail participants had a threefold risk (OR 3.18, 95% CI = 1.42–7.12) of hospitalisation compared to pre-frail ones. For participants *with* informal care available, the frail ones had a twofold higher risk of hospital admission compared to their pre-frail counterparts (OR 2.15, 95% CI = 1.66–2.78). This indicates a buffering effect of availability of informal care, however the difference was not statistically significant (*p* = 0.299). Regarding mortality, the data showed contradictory results: the availability of informal care is associated with an increased mortality risk for frail participants (OR 3.12, 95% CI = 2.23–4.37) compared to pre-frail participants, while this risk is only slightly higher for frail participants with no availability of informal care (OR 1.17, 95% CI = 0.29–4.74). Again this difference was not statistically significant (*p* = 0.278). Thus, in addition to the fact that none of the moderating effects were statistically significant, trends were inconsistent. Similar unexpected or inconsistent trends were found regarding educational level, sense mastery and self-management abilities.

For the outcome disability, two resources, income and living situation, showed a statistically significant interaction with frailty. However, the direction of these two moderating effects was opposite to that hypothesised. Among participants with a high income frail participants deteriorated more (Δ = 5.18 ± 7.88) than their pre-fail counterparts (Δ = 2.71 ± 6.39), while among those with a low income the changes in levels of disability were fairly similar between pre-frail and frail participants (about 3.0 points). Among those who were not living alone the mean change score was larger for frail than pre-frail participants (Δ = 4.72 ± 8.08 versus Δ = 2.64 ± 6.59 respectively). No large differences over time were detected between pre-frail and frail participants who were living alone (Δ = 3.16 ± 7.04 and Δ = 3.40 ± 7.96 respectively). The interacting effects of the other resources were not significant.

Results of all analyses were based on valid cases. For mortality, complete data were available. For hospitalisation, results were based on 1803 valid cases. Of the total number of missing cases (*n* = 617, 25% of the population) about one third can be explained by participants who were admitted to a nursing home (*n* = 53) or had died during follow-up (*n* = 132). The group with valid data was compared with the group with missing data on baseline characteristics using chi-square and Mann-Whitney tests. Participants in the group with missing data were significantly older, more often frail, less educated, more often living alone, had a lower sense of mastery, less self-management abilities and more (I)ADL disability at baseline. Similar patterns were found for the outcome disability (1883 valid cases); participants with missing data (*n* = 537) had less favourable baseline scores compared to valid cases.

## Discussion

The aim of the present study was to investigate whether specific resources moderate the impact of frailty level on adverse outcomes over a two-year period. Results show that frail older participants, compared to those who are pre-frail, have increased odds of mortality and hospitalisation, and deteriorate more on disability scores. This is in line with previous research [2, 23]. The resources studied have no moderating effects for the outcomes mortality and hospitalisation. Moreover, the directions of trends were inconsistent. Only for disability statistically significant moderating effects were found for the resources income and living situation. However, the direction of these results contradicted our expectations. Overall, we may conclude that the selected resources hardly seem to moderate the effects of the level of frailty on the adverse outcomes in our study.

Although previous research showed relationships between the resources we investigated and frailty [24], the resources were scarcely studied as moderating factors in the pathway from frailty to its adverse outcomes. Hoogendijk and colleagues investigated whether psychosocial resources (such as mastery and self-efficacy) moderate the effect of frailty on functional decline and mortality among community-dwelling older people [22]. They found no moderating effects of the resources they studied, including mastery, which is similar to the results of our study. In contrast, Dent et al. reported several moderating effects of psychosocial factors, including sense of control (mastery) [11]. This might be due to differences between the populations studied; Dent et al.’s study had a (smaller) hospital-based population including non-frail persons and had a larger proportion of frail people. Ament and colleagues found that educational level moderated the effect of difficulty in performing ADLs on self-perceived health in males and it moderated the effect of psychological distress on self-perceived health in women [12]. In our study no significant moderating effect of educational level was found. Ament et al. also found that living alone status (in women) significantly moderated the effect of difficulty in performing ADLs on receiving professional care. In their study, female participants who live with someone received less professional care, which is considered to be beneficial. We found that living with someone else leads to significantly more deteriorating disability scores compared to living alone, which is considered to be disadvantageous. Ament and colleagues defined frailty in a different way and used different outcome variables compared to our study, which might be reasons why the results seem to contradict. For all resources we found results which contradicted our expectations on one or more outcome measures. The effects of frailty status on the outcomes measures might be so dominant, that the moderating effects are being overshadowed.

The strength of the present study is that it is one of the first to investigate the moderating effect of several resources using longitudinal data of a large sample of community-dwelling pre-frail and frail older people. However, a relatively large proportion of outcome data were missing regarding hospitalisation and (I)ADL disability. As those with missing data had worse baseline scores, results of the present study should be interpreted with caution. Also, selective mortality or admission to a nursing home might have influenced the results, as the most severely frail participants dropped out for these reasons. It is uncertain to what extend and in which direction the missing data influenced the results.

So far, research on the moderating effect of resources is scarce and results vary between studies. In order to gain more insight into the role of resources in the pathway from frailty level to adverse outcomes, future research should try to include the frailest cases that were missed or had dropped-out in previous studies. Also, non-frail persons could be included as resources might have a beneficial effect especially in the early onset of frailty and less in the phases of pre-frailty and frailty. A follow-up period longer than two years might therefore also be necessary in order to find possible moderating effects. However, if moderating factors take many years to have beneficial effects, it is questionable if it is useful in daily practice to intervene in them, as frail people are already at risk of adverse outcomes. Furthermore, both the frailty criteria and the adverse outcomes (partially) that were used in this study have a physical nature. Their coherence is therefore fairly strong. Consequently, non-physical resources may not have a moderating effect. Given the latter two remarks, the focus of future research might be on (1) finding other moderating factors that are easy to intervene in and/or require less time to moderate, and hence are more useful in daily practice, and/or (2) choosing other (non-physical) outcome measures to study possible moderating effects of the resources.

## Conclusions

Results of the present study showed that frail older participants had increased odds of mortality and hospitalisation, and deteriorated more on disability scores, compared to those who were pre-frail. However, no clear moderating effects of the studied resources on the adverse outcomes associated with frailty were found among pre-frail and frail participants.

## Abbreviations

(I)ADL: (Instrumental) activities of daily living
CES-D: Center for epidemiologic studies depression scale
OR: Odds Ratio
SMAS-S: Self-management ability scale – short version
SQUASH: Short questionnaire to assess health-enhancing physical activity
TFI: Tilburg Frailty Indicator

## References

[1] Christensen K, Doblhammer G, Rau R, Vaupel JW (2009). Ageing populations: the challenges ahead. Lancet.

[2] Fried LP, Tangen CM, Walston J, Newman AB, Hirsch C, Gottdiener J, Seeman T, Tracy R, Kop WJ, Burke G, McBurnie MA (2001). Frailty in older adults: evidence for a phenotype. J Gerontol A Biol Sci Med Sci.

[3] Rockwood K, Mitnitski A (2007). Frailty in relation to the accumulation of deficits. J Gerontol A Biol Sci Med Sci.

[4] Mitnitski AB, Mogilner AJ, Rockwood K (2001). Accumulation of deficits as a proxy measure of aging. ScientificWorldJournal.

[5] Gobbens RJ, van Assen MA, Luijkx KG, Wijnen-Sponselee MT, Schols JM (2010). The Tilburg frailty indicator: psychometric properties. J Am Med Dir Assoc.

[6] Bouillon K, Kivimaki M, Hamer M, Sabia S, Fransson EI, Singh-Manoux A, Gale CR, Batty GD (2013). Measures of frailty in population-based studies: an overview. BMC Geriatr.

[7] de Vries NM, Staal JB, van Ravensberg CD, Hobbelen JS, Olde Rikkert MG, Nijhuis-van der Sanden MW (2011). Outcome instruments to measure frailty: a systematic review. Ageing Res Rev.

[8] Verbrugge LM, Jette AM (1994). The disablement process. Soc Sci Med.

[9] Op Het Veld LP, van Rossum E, Kempen GI, de Vet HC, Hajema K, Beurskens AJ (2015). Fried phenotype of frailty: cross-sectional comparison of three frailty stages on various health domains. BMC Geriatr.

[10] Hoeck S, Francois G, Geerts J, Van der Heyden J, Vandewoude M, Van Hal G (2012). Health-care and home-care utilization among frail elderly persons in Belgium. Eur J Pub Health.

[11] Dent E, Hoogendijk EO (2014). Psychosocial factors modify the association of frailty with adverse outcomes: a prospective study of hospitalised older people. BMC Geriatr.

[12] Ament BH, de Vugt ME, Koomen FM, Jansen MW, Verhey FR, Kempen GI (2012). Resources as a protective factor for negative outcomes of frailty in elderly people. Gerontology.

[13] Terstegge C, Houben T, Schefman S, Spee H, Hajema K, Mujakovic S, Quadvlieg M, Verberne N. BMC Geriatrics: Fried phenotype of frailty: cross-sectional comparison of three frailty stages on various health domains. Onderzoeksprotocol Limburgse monitor volwassenen en ouderen. GGD Limburg; 2012. doi:10.1186/s12877-015-0078-0. 10.1186/s12877-015-0078-0 PMC4496916 26155837

[14] Schroevers MJ, Sanderman R, van Sonderen E, Ranchor AV (2000). The evaluation of the Center for Epidemiologic Studies Depression (CES-D) scale: depressed and positive affect in cancer patients and healthy reference subjects. Qual Life Res.

[15] Bouma J, Ranchor AV, Sanderman R, Sonderen van E. BMC Geriatrics: Fried phenotype of frailty: cross-sectional comparison of three frailty stages on various health domains. Het meten van symptomen van depressie met de CES-D: een handleiding. Tweede herziene druk. UMCG/ Rijksuniversiteit Groningen: Research Institute SHARE; 2012. doi:10.1186/s12877-015-0078-0.

[16] Wendel-Vos GC, Schuit AJ, Saris WH, Kromhout D (2003). Reproducibility and relative validity of the short questionnaire to assess health-enhancing physical activity. J Clin Epidemiol.

[17] Statistics Netherlands (CBS) https://www.cbs.nl/en-gb/about-us/organisation. Accessed 31 Aug 2016.

[18] Bouman A, van Rossum E, Ambergen T, Kempen G, Knipschild P (2008). Effects of a home visiting program for older people with poor health status: a randomized, clinical trial in The Netherlands. J Am Geriatr Soc.

[19] Pearlin LI, Schooler C (1978). The structure of coping. J Health Soc Behav.

[20] Cramm JM, Strating MM, de Vreede PL, Steverink N, Nieboer AP (2012). Validation of the self-management ability scale (SMAS) and development and validation of a shorter scale (SMAS-S) among older patients shortly after hospitalisation. Health Qual Life Outcomes.

[21] Kempen GI, Miedema I, Ormel J, Molenaar W (1996). The assessment of disability with the Groningen activity restriction scale. Conceptual framework and psychometric properties. Soc Sci Med.

[22] Hoogendijk EO, van Hout HP, van der Horst HE, Frijters DH, Dent E, Deeg DJ, Huisman M (2014). Do psychosocial resources modify the effects of frailty on functional decline and mortality?. J Psychosom Res.

[23] Macklai NS, Spagnoli J, Junod J, Santos-Eggimann B (2013). Prospective association of the SHARE-operationalized frailty phenotype with adverse health outcomes: evidence from 60+ community-dwelling Europeans living in 11 countries. BMC Geriatr.

[24] Mello AC, Engstrom EM, Alves LC (2014). Health-related and socio-demographic factors associated with frailty in the elderly: a systematic literature review. Cad de Saúde Pública.

